# Minimal Intervention in Dentistry: A Literature Review on Biodentine as a Bioactive Pulp Capping Material

**DOI:** 10.1155/2021/5569313

**Published:** 2021-04-03

**Authors:** Naji Ziad Arandi, Mohammad Thabet

**Affiliations:** ^1^Department of Conservative Dentistry and Prosthodontics, Arab American University, Jenin, State of Palestine; ^2^Department of Orthodontics and Pediatric Dentistry, Arab American University, Jenin, State of Palestine

## Abstract

Root canal treatment has been the treatment of choice for carious pulp exposures. In the perspective of minimally invasive dentistry and preventive endodontics, a direct pulp capping procedure with a reliable bioactive material may be considered an alternative approach provided that the pulp status is favorable. However, the treatment of pulp exposure by pulp capping is still a controversial issue with no clear literature available on this topic, leaving the concerned practitioner more confused than satisfied. Biodentine is a relatively new bioactive material explored for vital pulp therapy procedures. This article discusses its role in direct pulp capping procedures. A thorough literature search of the database was done using PubMed, Google Scholar, and Scopus using the keywords preventive endodontics, calcium silicate cement, direct pulp capping, Biodentine, and vital pulp therapy. Reference mining of the articles that were identified was used to locate other papers and enrich the findings. No limits were imposed on the year of publication, but only articles in English were considered. This paper is aimed at reviewing the current literature on Biodentine as a direct pulp capping material. The review will provide a better understanding of Biodentine's properties and can aid in the decision-making process for maintaining the vitality of exposed dental pulp with minimal intervention.

## 1. Introduction

Preserving the vitality of the dental pulp is a key factor for long-term tooth survival. Vital pulpal therapy (VPT) is designed to preserve and maintain the vitality of the pulp tissue in a tooth that has been compromised by trauma, caries, or restorative procedure [1]. The objective is to stimulate the formation of tertiary dentine to retain the tooth as a functional unit. The procedures of VPT range from more conservative treatments including indirect pulp capping and direct pulp capping to more invasive treatments including partial pulpotomy and full pulpotomy.

Traditionally, direct pulp capping procedures were indicated only for young permanent teeth with traumatic and mechanical (iatrogenic) pulp exposures. The introduction of calcium silicate cements (CSCs) has caused a paradigm shift in the conservative treatment of deep caries and VPT. In the past, calcium hydroxide (CH) was the standard direct pulp capping material because it is capable of stimulating the formation of tertiary dentine [2]. However, it has shown major disadvantages by dissolution over time, the formation of tunnel defects beneath dentinal bridges, and poor sealing [3, 4]. Recently, CSCs including mineral trioxide aggregate (MTA) and Biodentine (Septodont, Saint-Maur-des-Fossès, France) have been suggested as alternative pulp capping materials [5, 6].

MTA was first introduced in 1993 [7]. The formula of this material was based on ordinary Portland cement and had tricalcium silicate, tricalcium aluminate, dicalcium silicate, and tetracalcium aluminoferrite as main constituents, in addition to bismuth oxide and calcium sulfate [8–10]. A white MTA variant was later introduced as an attempt to overcome the tooth discoloration caused by gray MTA. White MTA had a similar composition to gray MTA, but it had no tetracalcium aluminoferrite and had lower levels of aluminate [9, 11, 12]. In general, MTA had a few drawbacks, including long setting time, induction of tooth discoloration, and difficult handling.

Biodentine was introduced to the market in 2011 as a quick-setting bioactive dentine substitute. Biodentine is made up predominantly of highly purified tricalcium silicate (80.1%) as the main core material, calcium carbonate (14.9%) as a filler, and zirconium oxide (a radioopacifier) [13, 14]. The liquid contains water, calcium chloride (used as a setting accelerator), and a hydrosoluble polymer as a water-reducing agent [13, 14]. Biodentine does not contain calcium aluminate, calcium sulfate, or bismuth oxide like MTA. The absence of bismuth oxide in Biodentine is significant to its properties [15–17]; bismuth oxide present in MTA is known to retard setting [18], negatively influence biocompatibility [19], and cause discoloration [20].

Variations in the manufacturing process and absolute constituent ratios influence the clinical behavior of CSCs [21]. Biodentine has been reported to demonstrate better mechanical properties [13, 14, 22–31], better color stability [32, 33], less tooth discoloration [24], easier application, and a shorter initial setting time (12-16 min vs. 3-4 hours) than MTA [14, 23, 27–29, 34–37]. Its principal drawback is its low radiopacity [13, 14, 35, 37–39] and the difficulty to obtain the desired or bespoken consistency [40].  Table 1 summarizes the studies reporting the physicochemical and biological properties of Biodentine.

Studies investigating the attitudes and practices of dental practitioners towards VPT and management of deep carious lesions reported that the usage of Biodentine was quite low [69–71]. A study that investigated and compared the management of pulp exposures during removal of carious tissue by French, German, and Norwegian general dental practitioners reported that the majority of participants of the study in all three countries chose direct pulp capping treatment (68–92.7%). The majority of the respondents from France (55%), Norway (57%), and Germany (52%) chose CH as the pulp capping material of choice. Biodentine was preferred by 17% and 3% in France and Norway, respectively, while none of the respondents in Germany preferred to use it. On the other hand, partial pulpotomy was selected by a very low percentage (0.7-10%). Only 5% of the respondents in Norway chose Biodentine as the material of choice for partial pulpotomies, while none in Germany and France reported using it [69]. Chin et al. [70] investigated the attitudes and practices of VPT in permanent teeth with deep caries among dental practitioners within Wales. They reported low usage of Biodentine or MTA for vital pulp therapy. Cost and lack of training were the main key obstacles to the uptake of these materials. Croft et al. [71] studied the management strategies preferred by dental practitioners in Finland for a pulp exposed during carious tissue removal in adult patients. In the presence of asymptomatic pulpal exposure, 65% opted for direct pulp capping, 5% for partial pulpotomy, and 1% for coronal pulpotomy. In case of symptoms of reversible pulpitis, the distribution of management preferences was 42%, 10%, and 3%, respectively. The materials of choice for direct pulp capping, partial pulpotomy, or coronal pulpotomy were equally distributed between MTA (39%) and CH materials (40%). Biodentine was preferred by 19%, and the remaining 2% chose zinc oxide, eugenol, or other materials.

Biodentine may be a viable choice for conducting “preventive endodontics [72].” It may provide valid and less invasive modalities for root canal treatment, as well as predictable direct pulp capping procedures. This paper is aimed at reviewing the current literature on Biodentine as a direct pulp capping material. The review will provide a better understanding of Biodentine's properties and can aid in the decision-making process for maintaining the vitality of exposed dental pulp with minimal intervention.

## 2. Method

To conduct this review, Scopus, PubMed, and Google Scholar databases were used to search for peer-reviewed articles on direct pulp capping using Biodentine. Search terms used included “calcium silicate cement,” “direct pulp capping,” “Biodentine,” “pulp exposure,” “vital pulp therapy,” and “bond strength to Biodentine.” Reference mining of the articles that were identified was used to locate other papers to enrich the findings. The cross-referencing process went on until no new articles were identified. No limits were imposed on the year of publication, but only articles in English were considered.

## 3. The Setting Reaction and Bioactivity of Biodentine

### 3.1. Setting Reaction of Biodentine

**Figure 1:**
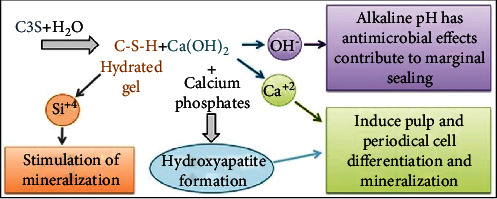
A diagram representing the setting hydration reaction of Biodentine. This reaction releases OH^−^, Ca^2+^, and Si^4+^ (reprinted and modified from Dental Materials, 35(1), T. Giraud, J. Charlotte, C. Rombouts, H. Bakhtiar, P. Laurent, I. About., Pulp capping materials modulate the balance between inflammation and regeneration, 24-35., 2019, with permission from Elsevier).

Biodentine sets with a hydration reaction. According to the manufacturer, an amalgamator is used for the trituration of the powder and liquid for 30 s. Once triturated, a paste of creamy consistency is obtained. The reaction of the powder with the liquid forms silicate hydrate gel and CH as a byproduct. CH dissociates into hydroxyl (OH^−^) and calcium ions (Ca^+2^), increasing the pH and Ca^+2^ concentrations [13, 17, 22, 24, 26, 29, 42, 44, 47, 48] (Figure 1).

Ca^2+^ released from CSCs promotes their bioactivity and apatite-forming properties [44, 73]. Ca^2+^ triggers the differentiation potential of dental pulp cells and facilitates mineralization leading to the formation of a dentine bridge upon the surface of the pulp in the long term [73, 74]. Biodentine has been reported to release significantly higher levels of Ca^2+^ than CH cements [17, 26], MTA [22, 29, 42], and TheraCal LC (Bisco Inc., Schaumburg, IL, USA) [48, 75]. Camilleri et al. [48] assessed the hydration of Biodentine and TheraCal LC after their application as pulp capping materials and compared it with direct hydration in an aqueous solution. They reported that Biodentine hydrated well and showed greater Ca^2+^ release than TheraCal LC. In another study, Fathy [75] studied the remineralization ability of Biodentine and TheraCal LC using a cell-independent model. The study concluded that Biodentine generated a significantly higher intensity of remineralizing elements in comparison to TheraCal LC. Biodentine's high Ca^2+^ release, as compared to CH cements and other CSCs, has been due to their solubility. The evident Ca^2+^ release of Biodentine has been attributed to their high solubility in comparison to CH cements and other CSCs [14, 22, 29, 35, 42–45].

An increase in Ca^2+^ release is also suggestive of OH^−^ release [26]. OH^−^ has been reported to increase the pH of the surrounding tissue [22, 37, 44, 47] and to provide for the antimicrobial effect of Biodentine [63–68]. An alkaline environment has been reported to trigger and promote the process of tissue repair [76–78]. The rise in pH leads to the formation of a thin layer of coagulation necrosis between the vital pulp tissue and the pulp capping material [68, 79]. The necrotic zone protects the underlying pulp cells from the alkaline pH of the material. A reparative dentinal bridge then begins to develop adjacent to the necrotic zone [80].

Biodentine has been also reported to release silicon ions (Si^+4^) into the adjacent dentine [81]. In general, Si^+4^ induce osteoblast proliferation and gene expression by involvement in metabolism, collagen synthesis, bone mineralization, and connective tissue cross-linking [82]. Si^+4^ released by Biodentine have been reported to promote mineralization and facilitate dentin bridge formation [79, 83, 84].

CSCs have the ability to form hydroxyapatite crystals after contact with phosphate-containing body fluid [85, 86]. The hydroxyapatite produced by Biodentine seals the tooth/material interface [13, 28, 49, 51–55, 87]. An excellent sealing capacity of the capping material is important for the success of the pulp cap; it seals the exposure site against bacterial microleakage [67, 88, 89] and provides a suitable environment for reparative dentin formation [90]. The literature reports that microbial leakage interferes with the pulpal response to capping materials. It stimulates the pulpal inflammatory activity and reduces the area of dentin bridge formation irrespective of the material used for pulp capping [89, 91, 92]. Biodentine has been reported to significantly reduce microleakage at the tooth/restoration interfaces by providing better secondary barriers under the surface seal [49, 54, 87].

### 3.2. Biodentine/Tooth Interface

The interaction of pulp capping materials with dentine is important as these materials need to protect the underlying pulp. According to the manufacturer's instructions, Biodentine is applied to the dentine surface without prior etching or bonding. The adhesion of Biodentine to dentine may result from the physical process of crystal growth within the dentinal tubules [93]. Hamama [94] evaluated the influence of dentine surface treatment with polyacrylic acid on the bonding of Biodentine to dentine and concluded that it can be applied directly to the dentine surface without any surface pretreatment. However, the application of 17% EDTA for 60 s has been reported to enhance the interaction of Biodentine with dentine [95].

The interaction at the interface between Biodentine has been assessed by various techniques and methodologies. Han and Okiji [81] observed the formation of a Biodentine/dentine interfacial layer. They also showed formation of tag-like structures extending from the material into dentinal tubules. Similarly, Chałas et al. reported the presence of an interfacial layer [96] and showed that Biodentine caused the uptake of Ca^2+^ and Si in the adjacent dentine. Atmeh et al. [97] also reported the formation of an interfacial layer “mineral infiltration zone” and a tag-like structure in Biodentine/dentine interface. They reported that the CSC's hydration produces an alkaline caustic effect, which degrades the collagenous component of the interfacial dentin. This in turn led to the formation of a porous structure which facilitated the increased permeability of high concentrations of calcium and carbonate ions. This leads to increased mineral deposition at the interface. Kim et al. [56] noted that the thickness of the interfacial layer at the Biodentine/dentine interface was significantly less than observed for MTA/dentine interface. Fathy [75] studied the remineralizing ability of Biodentine using a cell-independent model. The study reported that Biodentine was able to enrich the adjacent dentin area with significantly higher amounts of Ca^2+^that are essential for the remineralization process. It was also noted that the SEM images of the subsurface layer beneath Biodentine showed almost complete filling of intratubular dentin with mineralized tag or rod-shaped structures. Similarly, Hamama [94] reported a mineral-rich zone at the Biodentine/tooth interface and noted that Biodentine had a great affinity to exchange ions with tooth substrate and to bond chemically with dentine.

In contrast to the aforementioned studies reporting an “interfacial layer” or “mineral interfacial zone” at the Biodentine/dentine interface, Li et al. [98] reported no chemical changes nor tag-like structures at the Biodentine/dentine interface. Biodentine was noted to fill interfacial gaps by calcium phosphate deposition, however, without conducting chemical changes to the adjacent dentin. Recently, Hadis et al. [95] studied the interaction between Biodentine and dentine at their interface. They concluded that the interaction of Biodentine with the adjacent tooth structure was through the migration of Si into the dentine and deposition of phosphorus at the interface. They concluded that the “mineral infiltration zone” is nothing more than an artifact caused by specimen preparation for a confocal microscope.

### 3.3. Bioactivity of Biodentine

**Figure 2:**
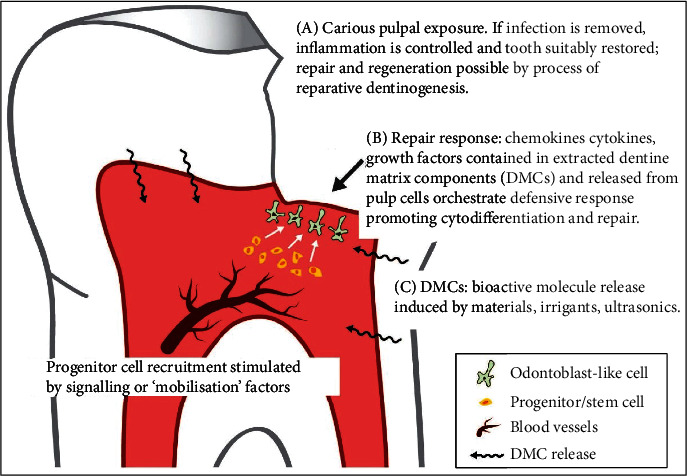
Reparative dentine formation involves a series of events in which a severe stimulus (e.g., pulp exposure) causes death of the primary odontoblast, which are then replaced following differentiation of progenitor cells into odontoblast-like cells under the regulation of bioactive molecules released from the dentine matrix. The bioactive molecules are released by caries, irrigants, and dental materials (e.g., MTA, Biodentine) (reprinted from International Endodontic Journal, 52(7), H. F. Duncan, P. L. Tomson, S. Simon, et al., Management of deep caries and the exposed pulp, 949-973., 2019, with permission from John Wiley and Sons).

Ideally, pulp capping materials should not only be inert (nontoxic to the pulp cells), but they should also be “bioactive” towards the tissues. In general, a bioactive material is defined as a material which has been designed to induce specific biological activities [99]. Based on this broad definition, biologically active materials may include those that promote tissue regeneration by stimulating migration, proliferation, and osteogenic differentiation of the cells. The growth of a layer of apatite on CSCs is an ideal environment for stem cell and osteoblast differentiation and colonization to support new hard tissue formation [19, 22, 47, 56–62]. Apatite together with the epigenetic signals correlated to ion release may well explain the bioactivity of Biodentine. It has shown the capacity to promote reparative dentinogenesis after pulp exposure, due to the enhanced regulation and modulation of bioactive molecules released from the dentine matrix, TGF-*β*1, and other growth factor secretion [55, 100, 101] (Figure 2). The release of TGF-*β*1 seems to induce a form of tertiary dentin; it attracts dental pulp stem cells to the Biodentine application (injury) site where it induces their differentiation into odontoblast-like cells secreting tertiary dentine under the material [60].

## 4. Direct Pulp Capping with Biodentine

A wide range of clinical indications have been published as case reports regarding the use of Biodentine. The use of Biodentine as a direct pulp capping agent has been reported [102–104]. Direct pulp capping is defined as the placement of a protective dressing directly over the exposed pulp to maintain pulp vitality by the formation of tertiary dentine [80, 105]. In certain clinical situations, direct pulp capping has a good prognosis, whereas, in others, failure is more predictable [4]. A traumatic or iatrogenic pulp exposure has a better prognosis than a carious pulp exposure [106]. Direct pulp capping lacks predictability in outcome when applied following carious pulp exposures because the pulp's response might be compromised by bacterial contamination and inflammation from progressing caries. However, success rates of direct pulp capping after carious exposures have been reported to depend on the technique and materials [106]. The introduction of newer bioactive materials might have made the procedure of direct pulp capping in teeth with carious exposure more predictable [107].

In general, direct pulp capping is aimed at maintaining pulp vitality and function. In the event of capping a pulp exposure, reparative dentine will form the mineralized bridge in an attempt to wall off the exposure site. At a cellular level, reparative dentine is believed to be produced following differentiation from undifferentiated mesenchymal cells and the formation of a new generation of odontoblast-like cells [108–111]. Other theories suggest that other cells such as fibroblasts may differentiate to produce the mineralized tissue [112]. Ricucci et al. [112] noted that in direct pulp capping of carious pulp exposures, the defects were repaired by the deposition of an amorphous dystrophic calcified tissue that closely resembled pulp stones rather than dentine. They added that fibroblasts and collagen fibrils, not odontoblasts or odontoblast-like cells, lined this atubular hard tissue.

### 4.1. Direct Pulp Capping Using Biodentine in Animal Models

Many studies evaluated the capacity of Biodentine, MTA, and CH in direct pulp capping of exposed pulp in animals [90, 100, 113–115]. They reported favorable results for direct pulp capping with Biodentine. However, it has been noted that there might be a major variation in the pulp tissue reaction between animals and humans [4]. This can be due to different metabolism and immune system responses [116]. Parirokh and Torabinejad [117] suggested that further work is required to investigate the clinical relevance of the results obtained from animal studies.

### 4.2. Direct Pulp Capping for Pulp Exposures in Caries-Free Permanent Teeth

Nowicka et al. [55] studied the pulp capping potential with Biodentine and MTA in pulp exposures in caries-free molars. They reported that both MTA and Biodentine had similar clinical results after 6 weeks of follow-up. Most of the teeth showed complete formation of the dentinal bridge and no inflammatory pulp response. Similar results were obtained by another study that assessed dentine bridge formation using CBCT imaging after direct pulp capping with CH, MTA, Biodentine, and a bonding agent (Single Bond Universal, 3M ESPE, Seefeld, Germany) in caries-free molars subjected to mechanical pulp exposures. The study found no significant difference between the MTA and Biodentine in terms of dentin bridge formation. However, the dentin bridges associated with Biodentine were reported to show the highest average and maximum volumes [118]. In a study by Jalan et al. [119], the histological response of the healthy pulp to Biodentine was evaluated and compared with CH (Dycal, Dentsply, Caulk, Milford, DE, USA) after 45 days. The teeth that were capped with Biodentine presented the formation of thicker and continuous dentinal bridges with less pulpal inflammation as a comparison to Dycal. Hoseinifar et al. [120] compared the histological response of human dental pulp after direct pulp capping with MTA, calcium-enriched mixture (CEM) cement, and Biodentine. They observed that the dentin bridge formation and the thickness of the dentin bridge formed in with Biodentine were higher than the other materials and that the amount of pulp inflammation was also higher in contact with Biodentine. The aforementioned studies investigating the pulp response to Biodentine were performed in caries-free teeth, thus undermining the generalizability of the results to teeth with carious exposures.

### 4.3. Direct Pulp Capping for Carious Pulp Exposures in Young Permanent Teeth with Immature Roots

Studies investigating direct pulp capping with various materials in young permanent teeth with immature root apices with carious exposures suggest that Biodentine might be a suitable capping material. Katge and Patil [121] compared the performance of Biodentine and MTA for direct pulp capping in young permanent molars (patients in the age group of 7 to 9 years). The study demonstrated 100% success with both Biodentine and MTA at 1 year of follow-up based on clinical and radiographic parameters. A study by Brizuela et al. [122] evaluated the clinical performance of MTA and Biodentine and compared them with CH for the management of permanent young teeth with caries in 7- to 16-year-old children with direct pulp capping (young molars with open and closed apices). The results of the study showed 100% success in the Biodentine group and 86.36% in each of the CH and MTA groups. There was no significant difference among the materials studied at all follow-up periods. However, the study noted that Biodentine offered some advantages over MTA (easy handling, sets in approximately 12 minutes, and does not cause discoloration of the tooth). Another study by Parinyaprom et al. [123] compared the success rates of direct pulp capping between two groups (MTA and Biodentine) in cariously exposed permanent teeth of 6-18 old patients. The sample of the study included teeth with carious exposures, irreversible pulpitis, early periapical involvement, and exposures up to 2.5 mm. They observed a success rate of 92.6% with MTA and 96.4% with Biodentine. The study concluded that irreversible pulpitis, carious exposure, early periapical involvement, or exposures up to 2.5 mm should not be considered as absolute contraindications for direct pulp capping.

### 4.4. Direct Pulp Capping for Carious Pulp Exposures in Permanent Teeth with Mature Roots

Direct pulp capping in mature permanent teeth with carious exposures has also been investigated [124–127]. These studies showed that vital permanent teeth, which are asymptomatic with cariously exposed pulp, can be successfully treated by direct pulp capping using Biodentine. A study by Linu et al. [124] investigated the outcome of direct pulp capping using MTA and Biodentine in mature permanent teeth (patients in the age group of 15 to 30 years) with carious exposures. This study showed that the success rates of MTA and Biodentine are 84.6% and 92.3%, respectively, with a follow-up period of 18 months after treatment. Radiographic findings of visible dentine bridge formation were observed in 69.2% and 61.5% of cases done with MTA and Biodentine, respectively. Similar results were reported by Hegde et al. [125]. They reported that over a period of 6 months, Biodentine and MTA showed 91.7% and 83.3% success rate, respectively. Awawdeh et al. [126] evaluated the clinical performance of Biodentine and white MTA (Angelus, Londrina, Brazil) in mature permanent teeth with carious exposure (patients in the age group of 16 to 51 years). They reported that Biodentine and MTA have similar survival probability (Biodentine = 91.7% and MTA = 96.0%) when used as pulp capping materials within the 3-year follow-up period. A study by Lipski et al. [127] evaluated Biodentine as a pulp capping agent in permanent teeth (patients in the age group of 11 to 79 years). In this study, the sample had pulps that were exposed during caries removal. The overall success rate after follow-up 1 to 1.5 years was 82.6%. This study noted that the success was age-dependent. A success rate of 90.9% was observed in patients younger than 40 years and 73.8% in patients 40 years and older.  Table 2 summarizes the studies reporting the results of direct pulp capping using Biodentine.

It seems that Biodentine might have good efficacy in direct pulp capping procedures in permanent teeth with both open and closed apices regardless of the nature of the pulp exposure being traumatic, mechanical, or carious. However, caution should be considered when recommendations are to be made on the use of Biodentine as an alternative to MTA and CH in direct pulp capping. Pulp capping procedures reported in the included studies were performed under different conditions, and also, different methods of evaluation (histological, clinical, and radiological) were used in the assessment. Furthermore, the success and prognosis depend on the age, type, site, and size of pulp exposure. The fact that MTA has been evaluated more extensively than Biodentine as a direct pulp capping material should be taken into consideration as well. Furthermore, as compared to MTA studies, the available Biodentine studies have a limited sample size.

## 5. Layering over Biodentine

The placement of a permanent, well-sealed restoration at the time of pulp capping is crucial to clinical success. A hermetic seal against bacterial infiltration is important to guarantee the successful outcome of the treatment [4]. Therefore, an adequate bond between the restorative material and the tooth, as well as between the restorative material and the pulp capping agent, is significant to ensure a proper and adequate seal and avoid clinical and radiographic failures. Biodentine, with its reduced setting time, may allow for resin composites and glass ionomer cements (GICs) to be layered over set Biodentine after 12 minutes, possibly allowing single-visit procedures.

### 5.1. Layering Composite Resins over Biodentine

When covered with a composite resin restoration, Biodentine is an appropriate dentine substitute [50]. The quality of the adhesive bond between Biodentine and the composite is of clinical significance in terms of the longevity of the restoration. Literature remains unclear concerning which adhesive agent performs better in combination with Biodentine. Some studies suggest the superiority of etch and rinse adhesives over self-etch systems [34, 128], while others report that self-etch adhesives provide higher bond strength [129], or that the choice of the adhesive strategy is irrelevant [130–133]. The differences in the recommendations made by the literature might be attributed to the differences in the study methodologies used and to the variations among the different resin composite brands and adhesive systems used in each study.

Cengiz and Ulusoy [128] noted that two-step etch and rinse adhesives (Prime & Bond NT, Dentsply DeTrey; Konstanz, Germany) showed higher bond strengths for composite restorations to Biodentine than did two-step etch adhesives (Clearfil SE Bond, Kuraray Noritake; Tokyo, Japan). Similarly, Meraji and Camilleri [34] reported that the two-step etch and rinse adhesive (Excite F; Ivoclar, Schaan, Lichtenstein) performed slightly better than the one-step self-etch adhesive (AdheSE One F, Ivoclar) in combination with Biodentine. On the contrary, different findings were presented by Çolak et al. [129]. They evaluated the shear bond strength of 3 different adhesives: Prime & Bond NT, Clearfil S^3^ Bond (Kuraray Medical, Osaka, Japan), Adper Prompt L-Pop (3M/ESPE, St. Paul, MN, USA) to Biodentine at 2 different time intervals: 9 min and 24 hours after setting. Adper Prompt L-Pop showed the lowest bond strength at both time intervals. The highest values were observed in Clearfil S^3^ Bond regardless of the time elapsed since its preparation.

However, others reported that the choice of the adhesive strategy was not that important for improving the reliability of the bond strength of a resin composite to Biodentine [130–133]. Odabas et al. [131] studied the resin composite bond strength when bonded to Biodentine with 3 different adhesive agents: Prime & Bond NT, Clearfil S^3^, and Clearfil SE. No significant differences were found between all of the adhesives at the same time intervals (12 min and 24 h). They concluded that the adhesive system did not affect the bond strength of Biodentine. However, they reported that the highest bond strength was obtained for the two-step self-etch adhesive (Clearfil SE) at a 24-hour period. Hashem et al. [130] reported no significant difference in bond strength when applying a universal adhesive (Scotchbond Universal, 3M ESPE, USA) to Biodentine either in the total-etch or self-etch mode. This was in agreement with the results obtained by Aksoy and Ünal [132]. They found no significant differences in the bond strength of the universal adhesives (Single Bond Universal, 3M ESPE, St. Paul, MN, USA., All-Bond Universal, Bisco Schaumburg, IL, USA., G-aenial Bond, GC, Tokyo, Japan) regardless of application mode (self-etch or etch and rinse). Carretero et al. [133] investigated the shear bond strength between Biodentine and a composite resin, using different types of adhesives (Optibond® FL, Kerr Corp, Orange, CA, USA., Solobond® M, Voco GmbH, Cuxhaven, Germany., Scotchbond Universal, 3M ESPE, St. Paul, MN, USA) at 2-time intervals (12 min and 24 h). The study reported that a statistically significant difference was found among the adhesives at 12 min, but not at 24.

The literature is also unclear regarding the proper timing to perform the definitive restoration. There is no consensus about the time after which Biodentine should be covered with the composite. Some studies suggest that the final composite restoration should be placed after at least 14 days to allow adequate setting and sufficient intrinsic maturation of the Biodentine [130, 132, 134–139]. Others claim that the final restoration is possible immediately after the application of Biodentine [129, 140, 141].

In general, when comparing the bond strength of Biodentine, TheraCal LC, and resin-modified glass ionomers (RMGI) to resin composites, Biodentine has been reported to have the lowest bond strength [128, 130, 136, 137, 142]. The higher bond strength for the RMGI and TheraCal LC might be attributed to the presence of hydroxyethyl methacrylate (HEMA), which promotes chemical adhesion to the resin-based restorative materials [143].

### 5.2. Layering GICs over Biodentine

Immediate layering with GICs is not recommended for Biodentine [34, 128, 144, 145]. Cantekin and Avci [144] evaluated the bond strength of a GIC (Fuji IX GC, Tokyo, Japan) to Biodentine 15 min after setting and reported that the GIC bonded to Biodentine at fairly low strength. Similarly, Meraji and Camilleri [34] reported a weak bond between Biodentine and GIC (Fuji IX). This was also in agreement with the results of another study [128], which evaluated the bond strength of Biodentine to RMGI (Fuji II, GC, Tokyo, Japan) and two GICs (Fuji IX) and (Equia Fil, GC, Tokyo, Japan) 12 min after setting. The study concluded that GICs and RMGI had inconvenient bond strengths to Biodentine. Meharwade et al. [145] evaluated the shear bond strength of the GIC (Fuji IX), RMGI (Fuji II), and resin composite with Biodentine as a base. According to the results, the bond strength of the resin composite to Biodentine was higher followed by RMGI and least with GIC. According to results of the fracture mode analysis reported in the aforementioned studies, the most observed fracture pattern between GIC and Biodentine was an adhesive failure with low bond strength [34, 128]. Tulumbaci et al. [146] reported that RMGI had low bond strength values to Biodentine. They noted that the low values might be attributed to the lower etching capability of RMGI. Nekoofar et al. [138] reported that a more stable and constant bond between RMGI and Biodentine could be achieved by delaying the layering procedure to 1 month and noted that this might be due to the improved chemical reaction between the carboxyl group of poly acids in GIC and Biodentine after maturation.

The acidity of GIC and RMGI may have a negative influence on immediate layering to the alkaline Biodentine. Studies have pointed out the negative effect of acidic pH on the properties of Biodentine and their hydration process [25, 147]. Elnaghy [25] evaluated the compressive strength, surface microhardness, morphologic microstructures, and bond strength of Biodentine and MTA after exposure to a range of acidic pH levels. They reported the occurrence of a substantial change in the microstructure of Biodentine and MTA after exposure to different pH values. Ashofteh Izadi et al. [147] analyzed the microstructure and crystalline structures of Biodentine when exposed to phosphate-buffered saline, butyric acid, and blood. They reported that acidic pH interfered with Biodentine's hydration. Furthermore, the acid-base reaction of GIC has a high affinity for water, so if it comes into contact with CSCs before their setting process is complete, it may uptake the water mixed with the CSCs, causing the setting process and properties to suffer [21]. However, the characteristics of the adhesion between Biodentine and GIC have not been elucidated. Further research will therefore be needed before a definitive conclusion can be reached regarding the bond between of GIC and Biodentine.

## 6. Conclusions

Recent research on the materials used in the management of pulp exposure supports less invasive strategies and highlights the role of CSC. Biodentine, a bioactive tricalcium silicate material, is not highly regarded or common among dental professionals. Yet, the literature is usually in favor of this material as a pulp capping material instead of MTA and CH. Biodentine has been reported to have good clinical efficacy in direct pulp capping procedures in permanent teeth with both open and closed apices. In exposed pulps, Biodentine tends to promote dentine bridge formation, and this may be due to a combination of its biocompatibility, alkalinity, and sealing ability. However, more long-term in vivo studies with a larger sample size and proper clinical settings are required to extend our knowledge and to draw a definitive conclusion on Biodentine.

## Figures and Tables

**Table 1:** Studies reporting the physicochemical and biological properties of Biodentine.

Material characteristics	Authors
Composition	Camilleri et al. [41], Grech et al. [14], Camilleri et al. [13]
Setting time	Alhodiry et al. [23], Jang et al. [27], Kaup et al. [35], Dawood et al. [29], Butt et al. [28], Villat et al. [36], Lucas et al. [37]
Radiopacity	Kaup et al. [35], Tanalp et al. [38], Corral et al. [39], Grech et al. [14], Camilleri et al. [13], Lucas et al. [37]
Solubility	Gandolfi et al. [42], Kaup et al. [35], Dawood et al. [29], Singh et al. [43], Abu Zeid et al. [44], Grech et al. [14], Al-Sherbiny et al. [22], Pushpa et al. [45]
Compressive strength	Elnaghy [25], Natale et al. [26], Jang et al. [27], Butt et al. [28], Dawood et al. [29], Govindaraju et al. [30], Grech et al. [14], Kayahan et al. [31], Al-Sherbiny et al. [22]
Hardness and flexural strength	Elnaghy [25], Alhodiry et al. [23], Grech et al. [14], Camilleri [46]
pH	Al-Sherbiny et al. [22], Gandolfi et al. [42], Abu Zeid et al. [44], Talabani et al. [47], Lucas et al. [37]
Calcium ion release	Al-Sherbiny et al. [22], Natale et al. [26], Li et al. [17], Dawood et al. [29], Gandolfi et al. [42], Camilleri et al. [48], Abu Zeid et al. [44], Talabani et al. [47]
Microleakage	Raskin and Dejou [49], Camilleri [46], Koubi et al. [50], Butt et al. [28], Bolhari et al. [51], Agrafioti et al. [52], El-Khodary et al. [53], Darsan et al. [54], Nowicka et al. [55]
Biocompatibility and bioactivity	Gomes-Cornélio et al. [19], Camilleri et al. [13], Talabani et al. [47], Kim et al. [56], Hasweh et al. [57], Araújo et al. [58], Athanasiadou et al. [59], Laurent et al. [60], Han and Okiji [61], Luo et al. [62], Al-Sherbiny et al. [22]
Antimicrobial activity	Poggio et al. [63], Ceci et al. [64], Bhavana et al. [65], Hiremath et al. [66], Koruyucu et al. [67], Estrela et al. [68]

**Table 2:** Clinical studies reporting the results of direct pulp capping using Biodentine.

Authors	Type of study	Number of teeth	Follow-up	Age (years)	Success rate^∗^
Studies investigating *pulp exposures in permanent caries-free teeth*					
Nowicka et al. [55]	RCT	MTA: 11 BD: 11	6 weeks	19-28	BD = 100% MTA = 100%
Nowicka et al. [118]	RCT	BD: 11 MTA: 11 CH: 11 Adhesive: 11	6 weeks	19-32	BD = 100% MTA = 100% CH = 100% Bond = 100%
Studies investigating *carious pulp exposures in young permanent teeth with immature roots*					
Katge and Patil [121]	RCT	MTA: 29 BD: 29	12 months	7-9	BD = 100% MTA = 100%
Brizuela et al. [122]	RCT	CH: 53 MTA: 56 Biodentine: 60	12 months	7-16	BD = 100% CH = 86.36% MTA = 86.36%
Parinyaprom et al. [123]	RCT	MTA: 27 BD: 28	6-54 months	6-18	BD = 96.4% MTA = 92.6%
Studies investigating *Carious pulp exposures in permanent teeth with mature roots*					
Linu et al. [124]	Retrospective	BD: 15 cases MTA: 15 cases	18 months	15-30	BD = 92.3% MTA = 84.6%
Awawdeh et al. [126]	RCT	BD: 34 MTA: 34	36 months	16-51	BD = 91.7% MTA = 96%
Lipski et al. [127]		BD: 112	12–18 months	11–79	BD = 82.6%
Hegde et al. [125]	RCT	BD: 12 MTA: 12	6 months	18-40	MTA = 91.7% BD = 83.3%

^∗^At the final follow-up recall. RCT: randomized controlled study. BD: Biodentine.
